# Targeted re‐sequencing confirms the importance of chemosensory genes in aphid host race differentiation

**DOI:** 10.1111/mec.13818

**Published:** 2016-09-15

**Authors:** Isobel Eyres, Ludovic Duvaux, Karim Gharbi, Rachel Tucker, David Hopkins, Jean‐Christophe Simon, Julia Ferrari, Carole M. Smadja, Roger K. Butlin

**Affiliations:** ^1^ Department of Animal and Plant Sciences University of Sheffield Western Bank, Alfred Denny Building Sheffield S10 2TN UK; ^2^ Edinburgh Genomics Ashworth Laboratories University of Edinburgh EH9 3JT Edinburgh UK; ^3^ Institut de Génétique, Environnement et Protection des Plantes, UMR 1349 IGEPP, Domaine de la Motte INRA 35653 Le Rheu Cedex France; ^4^ Department of Biology University of York York YO10 5DD UK; ^5^ Institut des Sciences de l'Evolution (UMR 5554 CNRS‐IRD‐CIRAD‐Université de Montpellier) Université de Montpellier cc065, Place Bataillon, Campus Triolet 34095 Montpellier Cedex 05 France

**Keywords:** *Acyrthosiphon pisum*, adaptation, chemosensory genes, genome scan, speciation, targeted resequencing

## Abstract

Host‐associated races of phytophagous insects provide a model for understanding how adaptation to a new environment can lead to reproductive isolation and speciation, ultimately enabling us to connect barriers to gene flow to adaptive causes of divergence. The pea aphid (*Acyrthosiphon pisum)* comprises host races specializing on legume species and provides a unique system for examining the early stages of diversification along a gradient of genetic and associated adaptive divergence. As host choice produces assortative mating, understanding the underlying mechanisms of choice will contribute directly to understanding of speciation. As host choice in the pea aphid is likely mediated by smell and taste, we use capture sequencing and SNP genotyping to test for the role of chemosensory genes in the divergence between eight host plant species across the continuum of differentiation and sampled at multiple locations across western Europe. We show high differentiation of chemosensory loci relative to control loci in a broad set of pea aphid races and localities, using a model‐free approach based on principal component analysis. Olfactory and gustatory receptors form the majority of highly differentiated genes and include loci that were already identified as outliers in a previous study focusing on the three most closely related host races. Consistent indications that chemosensory genes may be good candidates for local adaptation and barriers to gene flow in the pea aphid open the way to further investigations aiming to understand their impact on gene flow and to determine their precise functions in response to host plant metabolites.

## Introduction

Speciation depends on the evolution of barriers to gene flow, and natural selection is now considered to be an important driver in this process (Kirkpatrick & Ravigné 2002; Nosil *et al*. 2002; Via 2009; Nosil 2012); local adaptation can lead to reproductive isolation in the face of gene flow. Contact between populations that have undergone some divergence through selection or geographical isolation is a common occurrence, and the opportunity that this provides for gene flow may cause breakdown of the initial divergence. An important challenge in current speciation research is therefore to understand how lineages can maintain differentiation and progress towards speciation despite ongoing gene exchange (Smadja & Butlin 2011).

With the exception of polyploidy, speciation tends to be a long process, requiring the progressive build‐up of reproductive isolation (Abbott *et al*. 2013). Where lineages are undergoing ecological speciation in the face of gene flow, reproductive isolation can start with the action of divergent selection on locally adaptive loci. This initial divergence may then be facilitated by the association of local adaptation and assortative mating, by close linkage in the genome, by pleiotropy or where the same trait influences both components of isolation (Felsenstein 1981; Servedio 2008; Smadja & Butlin 2011). There is then the possibility that initially divergent genome regions will expand over time as gene flow diminishes between lineages (Feder *et al*. 2012).

One way to study the progress of barriers to gene flow and their role in contributing to speciation is to identify candidate loci in populations experiencing divergence based on local adaptation, early in the process of speciation, and to track the action of selection across a continuum of divergence through space or time (Jones *et al*. 2012; Martin *et al*. 2013). To do this effectively, we must be able to identify loci involved in the initial local adaptation with confidence. While many studies have identified highly differentiated loci that are potentially under divergent selection, successful follow‐up studies to outlier scans have rarely been achieved (Rogers & Bernatchez 2005, 2007; Wood *et al*. 2008; Butlin 2010; Jones *et al*. 2012; Malinsky *et al*. 2015).

Quantitative trait locus (QTL) studies have traditionally been used to identify genome regions connected to local adaptation (Hawthorne & Via 2001; Ungerer & Rieseberg 2003; Baxter *et al*. 2008), and population genomic scans for outlier loci are commonly used to identify outlier loci relating to local adaptation and the reduction of gene flow between populations (Nosil *et al*. 2008; Galindo *et al*. 2010). Both of these methods can now be performed with very large numbers of markers and therefore can have high resolution (Hohenlohe *et al*. 2010). However, it can still be a challenge to pinpoint the specific targets of selection with confidence; there are multiple reasons why outlier loci may be detected in one sample only, and it is important to confirm that identified outliers are the true targets of natural selection. Functional interpretation of differentiated, ‘outlier’, loci or QTL (Barson *et al*. 2015) and experimental tests for the action of selection (Barrett *et al*. 2008; Gompert *et al*. 2012) are ultimately critical but can be a major investment. Given the uncertainties associated with outlier detection (e.g. Hermisson 2009), an important step in many systems is to confirm outliers by repeating analyses in new samples, separated in time or space.

Where we have good reason to suspect the involvement of a gene category in a speciation system, targeted gene sequencing can allow us to look in more specific regions for signals of reduced gene flow, while avoiding some of the problems of the population genomics and QTL methods (e.g. false positives caused by multiple testing, uncertainty about the genuine target of selection) (Smadja *et al*. 2012). Combining QTL mapping with outlier loci scans, to associate outliers with phenotypes, can provide a powerful indication of the source of selection driving speciation (Rogers & Bernatchez 2005; Via & West 2008; Via 2012; Via *et al*. 2012) and reveals the enormous potential we now have to follow up on outlier scans once outliers have been confidently identified.

Host race formation in phytophagous insects represents an excellent model for the evolution of reproductive isolation resulting from divergent selection in the face of gene flow (Drès & Mallet 2002; Bush & Butlin 2004; Forister *et al*. 2011). Very high diversity is linked to specialization via host switching and co‐speciation in many insect taxa (Weiblen & Bush 2002). Host races (genetically distinct populations, locally adapted to different host plant species but still experiencing some level of gene flow), and their host plants (clearly defined species, but often geographically proximate), provide a very helpful set‐up for examining the interplay between divergent selection and ongoing genetic exchange (Drès & Mallet 2002).

The pea aphid, *Acyrthosiphon pisum*, is a well‐established model for the study of ecological speciation (Peccoud & Simon 2010) and was the first aphid species to have its genome sequenced (The International Aphid Genomics Consortium 2010). *A. pisum* lives and feeds on species of the bean family (Fabaceae); in Europe, at least 15 genetically distinct host plant‐associated populations (races) have been described, each associated with one or a few host plant species. *A. pisum* races show increased preference for and performance on their associated plant species in comparison with alternative host plants (Via 1991; Ferrari *et al*. 2008). Races form a continuum of divergence ranging from pairs which produce around 10% F1 hybrids up to and including strongly isolated host races with *F*_ST_ exceeding 0.8 in sympatry, which probably no longer experience gene flow (Peccoud *et al*. 2009, 2015). This continuum of divergence between races provides us with a rare opportunity to examine the progression of barriers to gene flow across the genome. Although pea aphid host plants have overlapping ranges (Peccoud *et al*. 2009), aphid host races both feed and mate on their specific plants, which leads to assortative mating and the potential for the evolution of reproductive isolation.

Because assortative mating is related to host plant, how aphids select a plant to settle and feed on has the potential to be an important component in the evolution of premating isolation. Indeed, the chemosensory system has frequently contributed to host, habitat and mate choice in a range of study systems (reviewed in Smadja & Butlin 2008). Aphid recognition of the host plant and establishment of phloem feeding has several stages (Powell *et al*. 2006; Simon *et al*. 2015); before an aphid settles to feed, it may respond to plant volatiles near the surface of the leaf (Nottingham & Hardie 1993) and undertake initial probing with the stylets (Caillaud & Via 2000). Volatile and nonvolatile odour and taste molecules are recognized in insects by a set of chemoreceptors found in the chemosensory organs (antennae, mouth parts and maxillary palps) (Kopp *et al*. 2008; Shiao *et al*. 2013). These chemosensory genes include gustatory (GR), odorant (OR) and ionotropic (IR) receptors (Hallem *et al*. 2006; Croset *et al*. 2010), as well as odorant binding proteins (OBPs) which are involved in the transport of odorants (Leal 2005), chemosensory proteins (CSPs) and sensory neuron membrane proteins (SNMPs) (Leal 2005; Jin *et al*. 2008; Vogt *et al*. 2009). Evidence is accumulating for the key role of chemosensory genes in host specialization in insects (Visser 1986; Whiteman & Pierce 2008; Schymura *et al*. 2010). They exist in large multigene families in most insects (Sánchez‐Gracia *et al*. 2009), and both their birth and death mode of evolution and the detection of positive selection on branches of these multigene families point to rapid evolution in specialized lineages (Matsuo 2008; Briscoe *et al*. 2013; Duvaux *et al*. 2015).

In the pea aphid, multiple lines of evidence now point to the importance of chemosensory genes as a category in underpinning feeding decisions. Behavioural studies indicate that aphids show a distinct preference for their associated host plant when presented with a choice of alternative hosts (Ferrari *et al*. 2006), as well as increased survival and fecundity. Genetic evidence from whole genome scans (Jaquiéry *et al*. 2012), targeted re‐sequencing (Smadja *et al*. 2012), examination of copy number variation (Duvaux *et al*. 2015) and gene expression (Eyres *et al*. 2016) has all found indications that chemosensory genes differ between pea aphid races. Although these studies confirm the value of further investigation of chemosensory genes in pea aphids and provide us with a set of potentially interesting target chemosensory genes, this type of broad genomic study is prone to problems of false positives, as well as questionable reliability and repeatability (François *et al*. 2016; Jensen *et al*. 2016). Before we progress to examine target genes in more detail, it is important to confirm the findings of these studies.

There is a large number of tests available for the detection of outliers relating to local adaptation (e.g. Beaumont & Nichols 1996; Beaumont & Balding 2004; Foll & Gaggiotti 2008; Whitlock & Lotterhos 2015). In general, these methods evaluate the genetic differentiation between populations and identify extreme values corresponding to candidate regions of the genome. Outlier scans have proved successful in many cases at identifying loci potentially under selection (Nosil *et al*. 2009; Butlin 2010). However, a disadvantage of many outlier detection methods is their requirement for a priori assignment of individuals to populations (Yang *et al*. 2012; François *et al*. 2016). In populations undergoing divergence in the face of gene flow, such as pea aphid host‐associated races, the potential for sampling migrants and hybrids is high, making confident assignment of individuals to populations a difficult requirement to fulfil. In this study, we use PCAdapt (Duforet‐Frebourg *et al*. 2014, 2015), a method for the detection of candidate loci using Principal Components analysis (PCA), which is individual‐based and therefore is well suited to analysing data where population level assignment of individuals is uncertain. As our interest lies in identifying loci relating to differences in host plant preference between aphids, rather than in analysing genetic population structure, it is useful to be able to identify outliers based on genetic divergence rather than a priori population classification. Because PCAdapt identifies factors underlying the major axes of genetic variation among individuals, and then searches for loci strongly influencing these factors, it also allows us to examine only the important variation among races, rather than all pairwise race comparisons, thus reducing the risk of false positives from multiple comparisons. Furthermore, unlike many model‐based outlier methods, PCAdapt does not assume an island model, and so is better suited to the wide range of levels of differentiation seen among pea aphid races.

Previous work (Smadja *et al*. 2012) has identified chemosensory genes as a promising set of candidate barrier loci; in an *F*_ST_ outlier scan of 9889 SNPs in 172 target genes (chemosensory and control), the proportion of outlier SNPs identified in Grs and Ors was significantly higher than in nonchemosensory control genes. Furthermore, this study identified a set of 18 chemosensory genes that were unusually divergent between host races. These chemosensory candidates were identified as outliers in comparisons between three of the more closely related, although still highly specialized, pea aphid races (feeding on *Medicago sativa*,* Trifolium pratense* and *Lotus pedunculatus*) (Ferrari *et al*. 2008, 2012; Peccoud *et al*. 2009) in a single geographical region.

In diverging populations, alleles underlying local adaptation can differ among localities because of drift, availability of mutations or differences in selection, but repeated patterns of differentiation across the geographical range of the pea aphid races would provide evidence for loci that diverge in response to common divergent selection pressures rather than as the result of stochastic processes. In addition, we wished to test whether loci involved in differentiation between one pair of races were also likely to contribute to differentiation between other pairs. Therefore, our intention here was to test the pattern of divergence in chemosensory genes across (i) a larger number of pea aphid races along the continuum of differentiation and (ii) multiple populations covering a broader geographical distribution. Incorporating a wider selection of aphid races, including the far more divergent races associated with *Lathyrus pratensis*,* Cytisus scoparius* and *Ononis spinosa*, will ultimately allow us to capitalize on the continuum of divergence in pea aphids, by examining patterns relating to the extent of divergence between races and the progression of barriers to gene flow across the genome. Additional races also potentially facilitate the identification of new chemosensory outliers relating to local adaptation in previously untested races. Repeating outlier scans on independently sampled aphids allows us to exclude false positives from the initial scan and confirm the association of outliers with host race, the target environmental variable. As argued above, this confirmation is likely to be a valuable step in many comparable studies.

## Materials and methods

### SNP data from capture sequencing

We used the capture sequencing data set generated in Duvaux *et al*. (2015) using SureSelect. This was generated from 120 aphids (between 12 and 17 individuals per host plant) from eight host plant species (*Lotus pedunculatus*,* Lotus corniculatus*,* Medicago sativa*,* Trifolium pratense*,* Lathyrus pratensis*,* Pisum sativum*,* Cytisus scoparius* and *Ononis spinosa*), sampled 30 m apart to ensure distinct genotypes (supplementary file 1a in Appendix S1, Supporting information). Aphids were collected in southeast England over three years, all less than 100 km apart. SureSelect, which uses RNA probes to capture regions of interest from genomic DNA, was used prior to sequencing. Capture targets were candidate genes potentially relating to identification and selection of host plants, including all of the chemosensory genes that had been partially or fully annotated in Assembly 1.0 of the pea aphid genome (Smadja *et al*. 2009; Zhou *et al*. 2010): 79 olfactory receptor (Or) genes, 77 gustatory receptor (Gr) genes, 11 odorant binding protein (OBP) genes and 10 chemosensory protein (CSP) genes. Other genes potentially involved in chemosensation, 11 ionotropic glutamate receptor (Ir) genes and 9 sensory neuron membrane protein (SNMP) genes, were also included as targets, along with putative cis‐regulatory regions relating to all these genes (for Ors, Grs, OBPs and CSPs, 50‐bp predicted regions were identified upstream of target genes and for IRs and SNMPs, 500‐bp upstream regions were targeted, details in Duvaux *et al*. (2015)). Sixty‐nine genes from the P450 gene family (Zhang *et al*. 2010) potentially relating to detoxification, five pheromone synthesis genes and five salivary protein genes were also included. Two hundred and eleven randomly chosen genes were added as controls. After mapping reads to the pea aphid genome (assembly 2.1) using stampy 1.0.17, 21610 SNPs were called for all 120 aphid genotypes using platypus 0.7.9.2 (Rimmer *et al*. 2014).

### PCAdapt analysis of capture sequencing data

Capture data were filtered, removing SNPs based on the following criteria: quality score < 40, copy number ! = 1, minor allele count < 3, scored in <60% of individuals, observed heterozygotes >10 more than expected. Removing three individuals without copy number information (Med210, Lped84, Lped82), along with duplicate clone Pisum5, left 7232 loci in 116 individuals.

These 7232 SNPs were analysed using the rapid, PCA‐based method in PCAdapt (version 3.0 in r version 3.2.4) (Duforet‐Frebourg *et al*. 2015). PCAdapt performs scans for natural selection using principal component analysis; examining correlations between SNPs and each principal component allows the detection of SNPs that strongly influence patterns of variation and are putatively involved in adaptive differentiation along these axes. PCAdapt does not require any prior definition of populations.

An initial run with *K* = 20 principal components was used to select the correct K; a scree plot indicated *K* = 7 (supplementary file 1b in Appendix S1, Supporting information) as appropriate. After running with *K* = 7, it was apparent that aphids in the *La. pratensis*‐associated race have a large number of very influential SNPs in PC1 (supplementary file 1c and 1d in Appendix S1, Supporting information); as this makes outlier identification difficult, we excluded *Lathyrus*‐associated individuals from subsequent analyses. Excluding *Lathyrus‐*associated individuals left 104 genotypes in seven races. We then reran PCAdapt with one fewer principal component (*K* = 6) (supplementary file 1e in Appendix S1, Supporting information). Component‐wise outlier scans were performed in PCAdapt, using loadings as the test statistic (corresponding to the correlation between each SNP and the principal component of interest), *P‐*values were calculated based on making a Gaussian approximation for each PC and estimating the standard deviation of the null distribution, see Duforet‐Frebourg *et al*. (2015), and after converting *P*‐values to *q‐*values, SNPs with *q *≤ 0.05 were considered ‘outliers’. If outliers are randomly distributed in the genome, as might be expected for false positives, the number in any one gene will follow a Poisson distribution. The poisson.test() function in r was used to identify ‘outlier’ genes containing significantly more SNPs with *P *≤ 0.05 than expected by chance, given the overall proportion of outliers and the total number of SNPs per gene, for each principal component in turn (the same strategy as used by Smadja *et al*. 2012). Loci with few SNPs but with a high proportion of outliers may not depart significantly from the Poisson expectation. Therefore, this test may be prone to false negatives, but it is expected to provide a conservative list of genes with strong differentiation.

### Aphid collection and DNA extraction for GoldenGate SNP genotyping

Pea aphids were collected from the same eight host plants as used in the capture sequencing data set: *La. pratensis*,* O. spinosa*,* C. scoparius*,* Lo. corniculatus*,* Lo. pedunculatus*,* P. sativum*,* M. sativa* and *T. pratense*. In the UK, collection took place over two years (2012 and 2013) in locations near Bristol, Peterborough, Sheffield and the Blankney estate in Lincolnshire. Aphids from mainland Europe were collected in France (Mirecourt, Volgesheim, Ranspach and Bugey) and Switzerland. Where possible aphids were included from at least two UK locations and two locations in mainland Europe. In total, 29 location‐ and race‐specific groups of aphids were included, with a minimum of two and a maximum of seven sampling locations per race, and a mean of 13.5 individuals per race per sampling location. Details of sampling locations can be found in supplementary files 1f and 1g in Appendix S1 (Supporting information).

Aphids were grown up clonally from field‐collected individuals on *Vicia faba* in the laboratory to provide enough individuals for DNA extraction. Aphids were stored in ethanol prior to DNA extraction. DNA was extracted from five aphids per genotype, using NucleoSpin Tissue Kit standard protocol (Macherey‐Nagel, Düren, Germany).

### GoldenGate SNP assay design, sample processing and allele calling

Target SNPs were identified in control, chemosensory and detoxification genes in the capture sequencing data set. To design our custom set of 384 SNPs, flanking sequences of 100 bp to either side of target SNPs were processed using the Illumina Assay Design Tool (ADT) in order to confirm their suitability for the assay, and the finalized panel of 384 SNPs was ordered from Illumina (Illumina, San Diego, CA, USA). The final 384 SNPs comprised 222 in chemosensory genes, 71 in nonchemosensory genes of interest (P450s, PS and Rad51C) and 91 in control genes. One hundred and twenty‐seven target SNPs were in genes identified as having a significant excess of outlier SNPs in the capture sequencing data set. SNP IDs, chromosome positions and flanking sequences for the panel of 384 SNPs are available in Table S1 (Supporting information).

SNP data were analysed from each plate in turn using the Genotyping module of Illumina's GenomeStudio package (Illumina). SNPs were filtered for quality using standard thresholds, SNPs with no polymorphism, SNPs with no heterozygotes and SNPs with indication of copy number variation were also removed. This left 179 high‐quality SNPs for further analysis (Table S2, Supporting information). Aphids with more than 12 null SNP calls were removed from the data set, leaving data for 373 aphids.

### PCAdapt analysis of GoldenGate SNP genotyping data

The 179 SNPs in 373 aphids were analysed using the rapid, PCA‐based method in PCAdapt (version 3.0 in r version 3.2.4; Duforet‐Frebourg *et al*. 2015). As with the capture sequencing data, an initial run (*K* = 20 principal components) indicated *K* = 7 as appropriate (supplementary file 1h in Appendix S1, Supporting information). As with the capture sequencing analysis, *La. pratensis*‐associated individuals had a large number of influential SNPs in PC1, so were excluded from subsequent analyses (supplementary file 1i in Appendix S1, Supporting information).

Excluding individuals from the *Lathyrus‐*associated race (PC1 score > 0.1) left 338 genotypes in seven races. We then reran PCAdapt with one fewer principal component (*K* = 6) (supplementary file 1j in Appendix S1, Supporting information). Component‐wise outlier scans were performed in PCAdapt, using loadings as the test statistic (corresponding to the correlation between each SNP and the principal component of interest), and after converting *P‐*values to *q‐*values, significant SNPs (*q* < 0.05) were considered ‘outlier SNPs’.

### Comparing within‐ and between‐race variation in GoldenGate SNP genotyping data

Based on clustering of aphid genotypes in PCAdapt, the race of 10 aphids was re‐assigned from the collection host to the typical host of the genetic cluster to which they belonged (presumed migrants sampled on nonhost plants), and six aphids were removed as potential early‐generation hybrid individuals (details in supplementary file 1k in Appendix S1, Supporting information). We then used locus‐by‐locus analysis of molecular variance (amova), performed in arlequin (v 3.5) (Excoffier & Lischer 2010), to examine hierarchical genetic structuring, by race and by locality within race, at each of the 179 GoldenGate SNP genotyping loci.

## Results

### PCAdapt analysis of capture sequencing data: clustering of individuals

**Figure 1:**
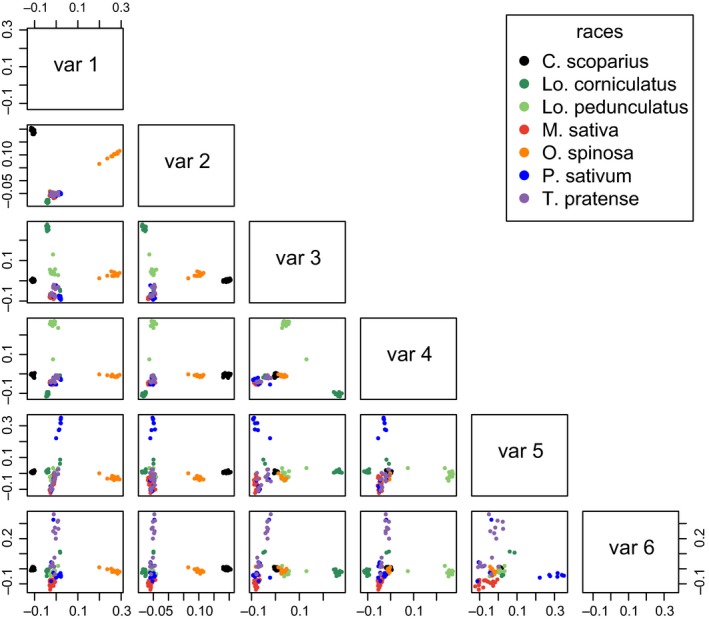
PCAdapt scores for all pairwise combinations of principal components 1 to 6, after excluding *Lathyrus pratensis‐*associated aphids (*K* = 6). Analysis based on 7232 SNPs from 104 aphid genotypes in seven host‐associated aphid races.

Running PCAdapt on the capture sequencing data set without *La. pratensis* individuals, with *K* = 6, allowed us to define six principal axes of variation (Fig. 1). The first principal component separates the *O. spinosa‐*associated individuals in one direction, and (to a lesser extent) *C. scoparius‐*associated individuals in the other, from all other races. PC2 separates *C. scoparius* and *O. spinosa*‐associated individuals from each other and from all other races. PC3 maintains the three most closely related races in our sample (*T. pratense*,* P. sativum* and *M. sativa*) in a single group and separates *Lo. pedunculatus, O. spinosa* and *C. scoparius*, and *Lo. corniculatus‐*associated clusters. PC4 separates both *Lotus‐*associated aphid races (*Lo. corniculatus* and *Lo. pedunculatus*) from all others. PC5 separates half of the *P. sativum‐*associated individuals from the other races, and PC6 separates half of the *T. pratense‐*associated individuals from all other races. *M. sativa‐*associated aphids are slightly separated from others in both PC5 and PC6. On the basis of these six axes, we can therefore distinguish aphids from the more divergent races, as well as some individuals (but not all) from the two highly similar races of *T. pratense* and *P. sativum*.

*F*_ST_ distributions (supplementary file 1m in Appendix S1, Supporting information), calculated according to groupings defined by the *La. pratensis‐*associated principal component and by the other six principal components (PC1–6), showed a large number of SNPs with *F*_ST_ = 1 in both the *La. pratensis* and the *C. scoparius‐*associated axes of variation, in agreement with PCAdapt findings. As expected, mean *F*_ST_ was lower in later principal components. The later principal components, which separate the *Medicago*,* Trifolium* and *Pisum*‐associated races studied by Smadja *et al*. (2012), show *F*_ST_ distributions compatible with the values previously reported (multilocus *F*_ST_ = 0.019–0.084).

### PCAdapt analysis of capture data: outlier analysis

Component‐wise outlier scans were performed in PCAdapt, using loadings as the test statistic (corresponding to the correlation between each SNP and the principal component of interest), *P‐*values were converted to *q*‐values to control for false discovery rate, and SNPs with *q *≤ 0.05 were considered ‘outlier SNPs’. A total of 503, 557, 299, 274, 111 and 5 SNPs with *q *≤ 0.05 were identified in PC1 to PC6, respectively. These correspond to SNPs with the highest loadings in each component; that is, they are the most influential SNPs in each axis of variation. Loadings, *P*‐values and *q‐*values of SNPs can be found in Tables S3, table 1 (Supporting information). A significantly higher proportion of outlier SNPs were in chemosensory genes than in nonchemosensory genes in principal components 1–4 (*z*‐tests for equality of proportions: PC1 control = 0.138, chemosensory = 0.168, *P* = 0.018; PC2 control = 0.147, chemosensory = 0.192, *P* = 0.001; PC3 control = 0.068, chemosensory = 0.127, *P* = 2e‐07; PC4 control = 0.072, chemosensory = 0.101, *P* = 0.004). Six hundred and fourteen significant global outliers were identified using the Mahalanobis distance (*q‐*value ≤ 0.05), and again a significantly higher proportion of outlier SNPs were in chemosensory genes than in nonchemosensory genes (control = 0.160, chemosensory = 0.248, *P* = 1e‐09).

**Figure 2:**
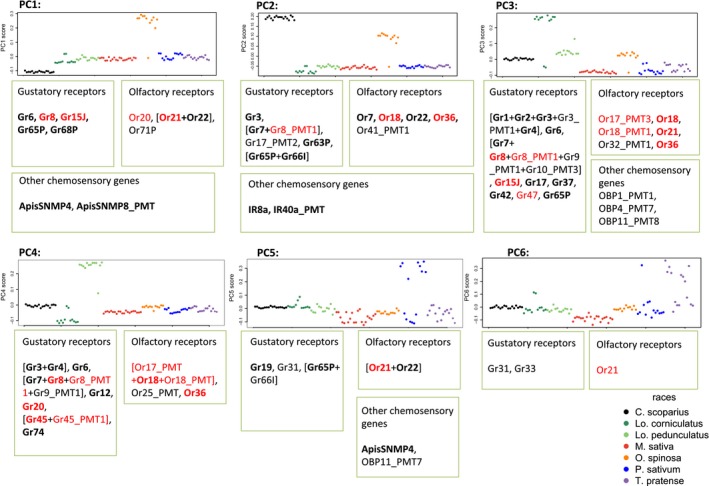
Loadings for each aphid genotype plotted for each principal component in turn. Outlier genes (Poisson test, *P* < 0.05) in boxes are associated with each principal component in the capture sequencing data set (7232 SNPs, 104 aphid genotypes, seven host‐associated aphid races). Genes on the same scaffold (pea aphid genome V2.1) are bracketed together, genes with >2 outlier SNPs are in bold, and genes identified as outliers in Smadja *et al*. (2012) are in red.

For each principal component in turn, and in the global analysis, genes were then considered ‘outlier genes’ when they contained significantly more SNPs with *q *≤ 0.05 than expected by chance (Poisson test), giving: 25 outlier genes in PC1, 24 in PC2, 35 in PC3, 29 in PC4, 15 in PC5 and 5 in PC6, of which 11, 14, 26, 17, 8 and 3, respectively, were chemosensory (Fig. 2; Table S3, table 2, Supporting information). Thirty‐five outlier genes were identified in the global analysis, of which 18 were chemosensory. Outlier counts and significance test values for all genes can be found in Table S3, table 2 (Supporting information). The majority of chemosensory outlier genes identified in each principal component were receptor genes.

Chemosensory outlier genes tended to be identified in blocks of close similarity and physical distance (Fig. 2); for example, gustatory receptors Gr1‐Gr4 (all present on scaffold GL350420) are all outliers in PC3, and where putative promoters were also identified, they were often present as outliers along with their downstream gene; for example, Or18 and an Or18 putative promoter regions are both outliers in PC3, and Gr8 and Gr45 are both outliers in PC4 along with their putative promoter regions. Positioning of scaffolds on a linkage map would provide a more robust understanding of the proximity of these outlier genes in the genome. Of the 18 outlier genes identified by Smadja *et al*. (2012) (*P* < 0.05, three or more outlier SNPs per gene), 14 were present in the filtered capture sequencing data set, and nine were confirmed as outliers in this new eight‐race comparison (Or17, Or18, Or20, Or21, Or36, Gr8, Gr20, Gr45 and Gr47), along with Gr15 (*P *< 0.05 but with <3 outlier SNPs).

### SNP data from GoldenGate SNP genotyping

After removing low‐quality SNPs and individuals (see Materials and methods), we were left with 391 unique aphid genotypes sampled from eight host plants, from between two and seven sampling locations per race, distributed across the UK, France and Switzerland. The retained set of 179 SNPs included 10 in SNMP genes (four genes), 46 control SNPs (44 genes), 43 in Grs (22 genes), nine in IRs (three genes), 31 in Ors (23 genes), 34 in P450 genes (24 genes), one in a CSP gene, one in an OBP gene, two in a PS gene and two in Rad51C, a control gene identified as an outlier by Smadja *et al*. (2012) (detail in Tabls S2, Supporting information).

### PCAdapt analysis of GoldenGate SNP genotyping data: clustering of individuals

**Figure 3:**
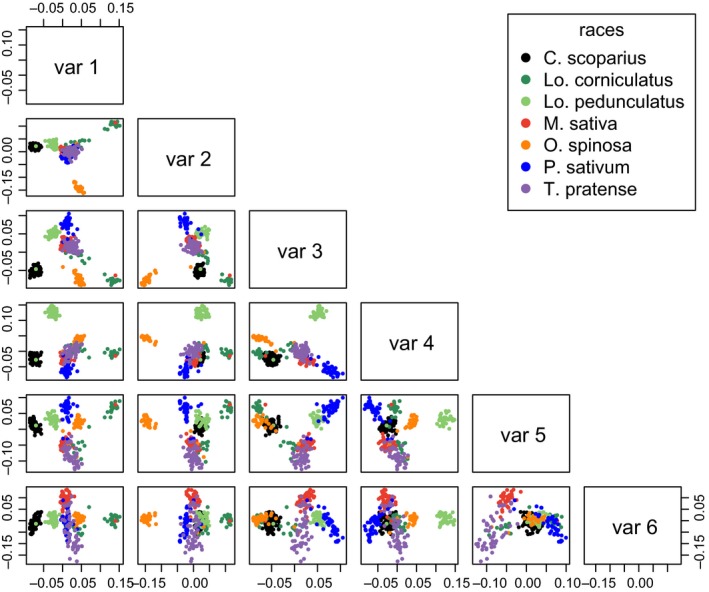
PCAdapt scores for all pairwise combinations of principal components 1 to 6, after excluding *Lathyrus pratensis‐*associated aphids (*K* = 6). Analysis based on 179 GoldenGate SNPs from 373 aphid genotypes in seven host‐associated aphid races.

Running PCAdapt on the GoldenGate SNP genotyping data set after excluding *La. pratensis*‐associated individuals, with *K *= 6, allowed us to define six principal axes of variation (Fig. 3). The first principal component separates half of the *Lo. corniculatus* individuals in one direction, and the *C. scoparius*‐associated individuals in the other direction, from all other races. PC2 separates *O. spinosa*‐associated individuals in one direction, and half of the *Lo. corniculatus* individuals in the other direction, from all other races. PC3 separates *O. spinosa‐*,* Lo. corniculatus*‐ and *C. scoparius*‐associated individuals in one direction, and *P. sativum‐*associated individuals in the other, from all other races. PC4 separates the *Lo. pedunculatus* race from all others. *T. pratense‐* and *M. sativa‐*associated individuals consistently have the most negative values in axis 5 and are separated from the other races in opposing directions in PC6.

Apart from individuals sampled from *Lo. corniculatus*, which broadly split into two clusters based on whether they were sampled in the UK or in mainland Europe on all axes (supplementary figure 1l in Appendix S1, Supporting information, Fig. 3), individuals tend to fall into groups on the basis of host plant association and not on the basis of geography. A number of individuals in the GoldenGate SNP genotyping data set appeared to be migrants; that is, they were collected on one plant species, but are genetically most similar to aphids collected from a different host (e.g. two individuals sampled on *La. pratensis* cluster with other races, one with *Lo. corniculatus‐*associated individuals, and one with *P. sativum*‐associated individuals). A number of individuals may also be hybrids between two races, as they fall into different host‐associated clusters on different axes of variation (i.e. they have some SNP alleles typical of one race and other SNP alleles typical of a different race, e.g. one individual collected from *C. scoparius* clusters firmly with *O. spinosa‐*associated individuals in PC4). Although aphids from *T. pratense* and *M. sativa*, two of the most closely related races are not so discretely separated, all other races form distinguishable clusters on at least one principal component.

### PCAdapt analysis of GoldenGate SNP genotyping data: outlier analysis

Component‐wise outlier scans were performed in PCAdapt, using loadings as the test statistic (corresponding to the correlation between each SNP and the principal component of interest), *P‐*values were converted to *q‐*values, and SNPs with *q *≤ 0.05 were considered ‘outlier SNPs’. A total of 14, 17, 16, 5, 2 and 1 SNPs with *q *≤ 0.05 were identified in PC1 to PC6, respectively. These correspond to SNPs with the highest loadings in each component and are the influential SNPs in each axis of variation. Loadings, *P‐*values and *q‐values* of SNPs can be found in Table S2 (Supporting information). Of these 55 outlier SNPs, 42 (76%) are in chemosensory genes, while only three (5%) are in control genes.

### arlequin analysis of GoldenGate SNP genotyping data

The amova analysis revealed that a large percentage of total genetic variation was between the eight host‐associated races (47.79%, *P* < 0.005), while a much smaller percentage of total variation was attributable to between‐locality differences within each race (5.63%, *P* < 0.005). Examining the mean percentage of total genetic variation explained by among‐group and between‐geographical‐location variation in the locus‐by‐locus analysis allowed us to compare chemosensory and control SNPs. Among‐group variation was lower (20.88%) and between‐location variation was higher (8.18%) in control loci in comparison with chemosensory loci (45.80% and 6.90%, respectively), demonstrating the importance of between‐race differences in chemosensory genes in comparison with neutral loci.

### Comparison of capture sequencing and SNP genotyping results

The capture sequencing data set contained far more SNPs (7232), examined in fewer individuals (116) in eight races sampled in close proximity, while the GoldenGate SNP genotyping data set contained fewer SNPs (179), examined in a larger number of individuals (391) and covering multiple populations from a far larger European sampling distribution. Nevertheless, the axes identified in our capture sequencing and SNP genotyping data sets are broadly equivalent, although the order differs between analyses (as might be expected from the different composition of the samples): PC1 in the capture sequencing analysis and PC2 in the GoldenGate SNP genotyping analysis both distinguish *O. spinosa*‐associated individuals, PC2 in the capture sequencing and PC1 in the GoldenGate SNP genotyping analysis both distinguish *C. scoparius*‐associated individuals, while PC3 in the capture sequencing and PC1 in the GoldenGate SNP genotyping analysis both distinguish *Lo. corniculatus* individuals. PC4 separates the *Lo. pedunculatus‐*associated population in both analyses, and *M. sativa* and *T. pratense*‐associated individuals are distinguished by PC5 in both analyses. *P. sativum*‐associated individuals can be distinguished from other races in capture sequencing PC5 and GoldenGate SNP genotyping PC3.

SNPs with a high loading in their significant GoldenGate SNP genotyping component often have a high loading in the equivalent capture sequencing component; for example, the same SNP (Or36.1_17759) has the top loading in PC4 in both capture sequencing and SNP genotyping analyses, and the top SNP in the capture sequencing PC5 (Gr21.1_461003) has the 11th highest loading in GoldenGate SNP genotyping PC5. Of the 27 significant GoldenGate SNP genotyping outlier SNPs present in the capture sequencing data set, 23 also make a significant contribution to a capture sequencing factor. The four SNPs not contributing include one control SNP (Control_g84.3_29958), two SNPs in P450 genes (P450_g33.1_53017 and P450_g48.9_38206) and one Gustatory Receptor SNP (Gr1.2_96172). To simulate a null expectation for overlap between the two data sets, the total number of significant GoldenGate SNPs (34) was randomly re‐assigned to the set of 179 GoldenGate SNPs with 100 000 permutations, and for each permutation, we calculated the overlap between capture SNP significance at each SNP and the randomly assigned significant GoldenGate SNP. The real overlap of 23 SNPs significant in both data sets was significantly greater than this expectation (*P *< 0.0001).

**Figure 4:**
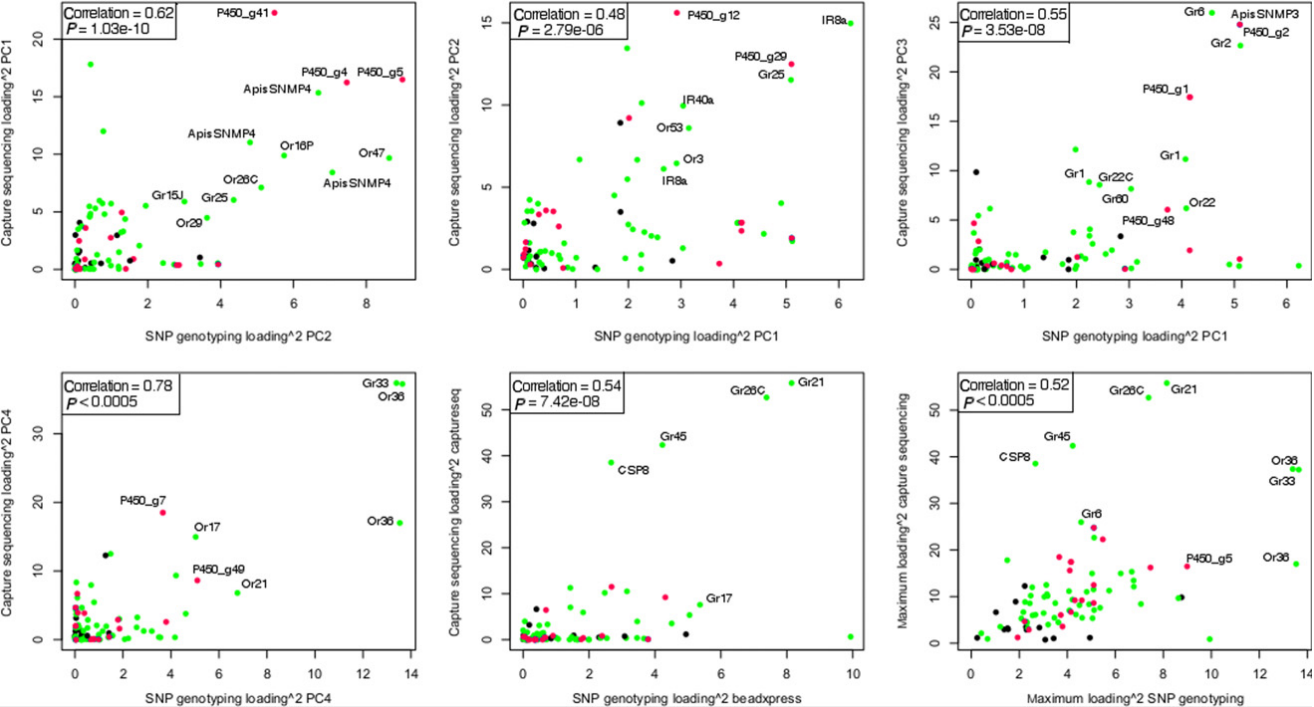
Squared loadings for SNPs in each principal component of the GoldenGate SNP genotyping data set plotted against squared loadings for the most strongly correlated principal component in the capture sequencing data set (left to right, top to bottom: capture PC1 vs. SNP genotyping PC2, capture genotyping PC2 vs. SNP genotyping PC1, capture PC3 vs. SNP genotyping PC1, capture PC4 vs. SNP genotyping PC4, capture genotyping PC5 vs. SNP genotyping PC5, and maximum squared loading capture genotyping vs. maximum squared loading SNP genotyping). Black = control, pink = P450, green = chemosensory.

There were significant, strong positive correlations between squared loadings of SNPs in the two data sets (Fig. 4), both when comparing broadly equivalent components (see Fig. 4), and when looking at maximum loadings per SNP across axes in each data set (Pearson's correlation = 0.52, *P < *0.0001).

There is substantial overlap in the genes identified between axes of variation: 389 outlier SNPs are related to more than one principal component in the capture sequencing data set. Furthermore, 14 chemosensory outlier genes (Poisson test *P *< 0.05) and five putative chemosensory promoter outliers (Poisson test *P *< 0.05) are identified in more than one principal component. In the GoldenGate SNP genotyping data set, 12 outlier SNPs are present in more than one axis of variation.

## Discussion

Pea aphids provide a promising system for examining the process of speciation with gene flow, and the progression from initial natural selection acting on adaptive loci to complete genomic differentiation and reproductive isolation between races. Chemosensory genes appear to be important targets of natural selection in this system; examining the differentiation of these genes between races, and how this changes as divergence between races increases, will enable follow‐up work looking at the genetic architecture of speciation with gene flow (making good use of the continuum of divergence between races seen in the pea aphid). Previous studies have indicated the value of further investigation of chemosensory genes in pea aphids. However, the types of large‐scale genomic study used to identify targets of natural selection are prone to false positives and have questionable reliability and repeatability (François *et al*. 2016; Jensen *et al*. 2016). Before we progress to examine target genes in more detail, it is important to confirm the findings of these studies. Here, we have undertaken a comprehensive follow‐up to previous work, over a broader geographical range than previously examined to confirm the presence of chemosensory outlier genes and their relationship to host plant adaptation. This is a step that could usefully be applied in many other comparable systems. We have also incorporated additional, more divergent host races giving insight into the role of the same genes at different stages in differentiation.

We have analysed genetic information from two data sets not previously used to detect outlier SNPs. The capture sequencing data set (Duvaux *et al*. 2015) included pea aphids from eight races: the three originally looked at by Smadja *et al*. (2012), and five more. In addition to confirming the repeatability of outliers among the original three races (Table 1), extending the outlier scans to additional races allowed us to test whether the same chemosensory loci were implicated in multiple host shifts. The GoldenGate SNP genotyping data set included pea aphids from the same eight races, this time sampled from locations across the UK, France and Switzerland. Sampling aphids from more localities across a broader geographical range enabled us to check that outlier genes relate directly to host plant species: there will have been other environmental variables correlated with race where only single geographical regions were examined, whereas replication across different localities and years tends to confirm the relationship between chemosensory gene differences and adaptation to host plants. Given ongoing gene flow among races (Peccoud & Simon 2010), consistent patterns of differentiation are unlikely to be explained by genomic regions of low recombination, whose effect on differentiation is greatest where gene flow is low or absent, but additional evidence for the action of divergent selection is still desirable (Jensen *et al*. 2016). Our results identify good targets for this future work.

**Table 1:** Outliers in each data set (Smadja *et al*., Capture Sequencing and GoldenGate SNP genotyping), for genes present in all data sets, two data sets and just one data set each. Smadja *et al*. (2012) and Capture sequencing outliers with *P *< 0.05 Poisson probability of the observed or a greater number of SNP outliers given the number of SNPs in the gene and the overall proportion of outliers. Outliers from GoldenGate SNP genotyping are genes containing a SNP with a significant loading (*q* < 0.05) in PCAdapt

	Analysed in		
	All 3 data sets	2 Data sets	1 Data set
Outliers in			
All 3 data sets	Gr15, Or21, Or36		
Smadja *et al*.+ capture	Gr45, Or17	Gr20, Gr47, Gr8, Or18, Or20	
Smadja *et al*. + SNP	Rad51C	—	
Capture + SNP	Gr1, Gr2, Gr3, Gr33, Gr4, Gr6, Gr9	ApisSNMP4_ref	
Smadja *et al*. only	Or29	Gr39, Or11, Or13, Or14, Or15, Or51, Or56	Gr59, Or6, Or61, Or62, Or73
Capture only	Gr17, Gr31, Gr65, Gr68, Or22, Or32, Or7	Gr10, Gr12, Gr19, Gr37, Gr42, Gr63, Gr66, OBP11, Or25, Or41, Or71, IR40a	Gr7, Gr74, OBP1, OBP4, ApisSNMP8_ref, IR8a
SNP only	Gr21, Gr25, Gr26, Or16, Or26, Or3, Or47	Gr60, ApisSNMP3_ref	—
Never an outlier	15	70	37

### Chemosensory genes confirmed as targets of selection

We were able to confirm the findings of Smadja *et al*. (2012) that a significantly higher proportion of outlier SNPs lie in chemosensory genes than in control genes and that this is true in different samples of aphids, from more races and localities. In both data sets analysed here, we again show that Gr and Or genes form the majority of chemosensory outlier loci. We specifically re‐identify ten of the outlier genes found in Smadja *et al*. (2012) in the capture sequencing data set (Table 1; Table S5, Supporting information), and three genes (Gr15, Or21 and Or36) were identified in the Smadja *et al*. (2012) analysis and in both the capture sequencing and GoldenGate SNP genotyping data sets. The correlation between the two analyses undertaken in this study was also strong; all chemosensory outlier SNPs identified in the GoldenGate SNP genotyping analysis (incorporating multiple populations per race), that were present in the capture sequencing data set, were also identified as outliers there.

By repeating outlier analyses on eight races, we confirm that differences in chemosensory genes are important to the divergence of the broader spectrum of pea aphid races, and incorporating more localities in our GoldenGate SNP genotyping data set allowed us to confirm a direct link between plant choice and chemosensory differences, distinct from other environmental variables that might be correlated with differences between single populations. amova showed large contributions of race and small contributions of locality to genetic variation, a pattern that was more pronounced in chemosensory than in control genes, supporting the relationship between chemosensory gene divergence and race in the face of gene flow. The congruence we observed between multiple independent samples of aphids provides support for the individual outlier genes identified, the general importance of chemosensory genes in between‐race differences and more specifically the potential role of Grs and Ors. Although the repeated identification of specific outlier loci could relate to underlying genomic architecture at these sites (Jensen *et al*. 2016) (which one would expect to be the same between aphids in different data sets), comparisons of gene categories are particularly informative indications of the validity of our results as there is little reason to expect that all chemosensory receptor genes will share an unusual feature such as a distinct mutation rate or low diversity, given that they are widely distributed in the genome.

ORs and GRs in insects tend to be activated in combinations to signal the presence of specific compounds (Hallem *et al*. 2006). As previously suggested (Smadja *et al*. 2012), mutations in these genes could potentially lead to changes in sensitivity or specificity of nerve activation, and combinations of mutations in different receptor genes might be required for a complex modification of response to multiple compounds differing between host plants. As ORs and GRs belong to large, fast evolving gene families, and are the main peripheral discriminators, they are the best a priori targets for involvement in host shifts. This assumption is supported by a number of studies highlighting the involvement of chemoreceptors in differences between host‐associated races (McBride 2007; Smadja *et al*. 2012; McBride *et al*. 2014; Duvaux *et al*. 2015). In contrast, OBPs and CSPs are smaller more conserved families, more involved in presenting ligands to receptors. Although they have been implicated in some host shift cases (Matsuo *et al*. 2007; Dworkin & Jones 2009), we find little evidence of their importance in pea aphid host race formation. Duvaux *et al*. (2015) and McBride (2007) both relate gain and loss of chemoreceptors to between‐race differences. Our finding of physical clusters of outlier genes (likely to result from recent tandem duplications; Smadja *et al*. 2009) may suggest that divergence after duplication is critical for the evolution of new response patterns (Fig. 2).

### The genetic architecture of divergence between races

Incorporating more races into our analysis has allowed us to identify a large number of new chemosensory genes as potential targets of selection. It is clear that the same set of genes is not necessarily involved in each adaptive host shift in the pea aphid; different chemosensory genes were outliers on different axes of variation although some axes separate multiple races. Identifying outlier genes in the more distinct aphid races, such as those very divergent in *O. spinosa* and *C. scoparius‐*associated races, will be useful for follow‐up work, as we cannot necessarily expect to examine divergence at the same chemosensory outliers in all race comparisons. The identification of multiple targets of selection relating to the same adaptive shifts suggests an polygenic basis of local adaptation in the pea aphid, fitting with the findings of Hawthorne & Via (2001), Caillaud & Via (2012) and Jaquiéry *et al*. (2012) who all identified multiple QTLs relating to aphid plant choice. The overlap in some cases between chemosensory genes identified on different axes of variation (see Table S5, Supporting information and Fig. 2) also shows how the same chemosensory genes can be involved in different adaptive host shifts, consistent with a combinatorial model of chemoreceptor activation (Hallem & Carlson 2004; Carey & Carlson 2011), and with the possibility that combinations or varying concentrations of plant compounds may act to trigger host acceptance or rejection.

Smadja *et al*. (2012) found that outlier genes could be divided into those with mainly nonsynonymous substitutions and those with mainly synonymous site substitutions, and took this to imply a role for regulatory changes in the loci with mainly synonymous outliers, the high divergence at synonymous sites reflecting divergent selection in closely linked regulatory regions. Consistent with this, we detected a large number of putative promoter regions as outliers. Often an outlier putative promoter was present upstream of a gene that was also identified as an outlier (e.g. Gr4, Gr8 and Or18 in PC3 and Gr45 and Gr8 in PC4 of capture sequencing data). This could be the result of hitchhiking in regions surrounding targets of selection, or it could relate to evolution of gene expression in some receptors. Although chemosensory genes as a class do not show more differential expression between races than other genes, some chemosensory genes are significantly differentially expressed between pea aphid races (Eyres *et al*. 2016); however, there is almost no overlap between these differentially expressed genes and the putative promoters identified here (ApisSNMP8 being the only exception).

The number of loci with extremely high loadings, equivalent to fixed differences, between *La. pratensis‐*associated aphids and the others was notably high, suggesting the possibility of the accumulation of extensive neutral divergence between the *La. pratensis*‐associated race and the other races. In accordance with this, outlier SNPs relating to *La. pratensis* contained a considerable number of control SNPs. Peccoud *et al*. (2009) suggested that the highly genetically differentiated *La. pratensis‐*associated race was nearing complete speciation, as no hybrid was detected with sympatric races, and our results are consistent with the minimal gene flow estimated between this more divergent race (Peccoud *et al*. 2009), in comparison with the higher gene flow between more genetically‐similar races, where loci experiencing barriers to gene flow will stand out more clearly against a background of low differentiation (Nosil *et al*. 2009; Butlin 2010). This pattern of increased neutral divergence in the race with the lowest ongoing gene flow supports the pea aphid host race system as a promising one for examining the genomic architecture of speciation with gene flow as races progress towards complete reproductive isolation.

### PCAdapt is a useful tool for analysing data with uncertain population assignment

PCAdapt needs no prior information about assumed population membership of samples. On the whole, aphids clearly clustered on the basis of the plant that they were collected from. However, we detected multiple possible hybrids, as well as migrant aphids that clustered with individuals sampled from a different host plant, in our large SNP genotyping data set. Because we were interested in identifying loci relating to differences in host plant preferences between races, using methods that require a priori knowledge of population structure would have required us to exclude or reclassify these individuals. Not doing this removed any artificial population structuring, which could arise from removing intermediate or nonconforming genotypes. In some cases (*P. sativum* and *T. pratense* in capture sequencing data and *Lo. corniculatus* in GoldenGate SNP data), PCAdapt was able to identify unexpected substructure within races, which are normally found to have little substructure, regardless of spatial scale (Frantz *et al*. 2006; Peccoud *et al*. 2008; Ferrari *et al*. 2012). The splitting of *P. sativum* and *T. pratense* individuals in the capture sequencing data is particularly interesting, as it was not identified in the analysis of the same data set (Duvaux *et al*. 2015) on the basis of 1777 SNPs and random forest clustering. Instead, Duvaux *et al*. identified two clusters of the *M. sativa‐*associated individuals. For the most similar races, presumably with the most recent origin and/or the highest gene flow, these findings may indicate that there is some overlap between spatial and host‐associated structure. This emphasizes the value of avoiding prior classification, especially where this is based on a small number of markers chosen for their ability to separate host races in a single region (as with microsatellites often used in pea aphid studies; Jaquiéry *et al*. 2012).

By looking at outliers relating to the principal components of genetic variation, rather than looking for global or pairwise *F*_ST_ outliers, we get a more biologically realistic insight into divisions between races, presumably looking at variation reflecting historical population subdivision by host switching, and reflecting true adaptive differences between relevant groups of races. This method also enabled us to carry out far fewer comparisons—with six principal components of variation rather than 28 pairwise comparisons between races—thus reducing the problems associated with multiple testing.

## Conclusions

The positive identification of outliers based on differences within and between populations can be caused by many factors other than divergent selection. Population size change (Teshima *et al*. 2006), population structure (Excoffier *et al*. 2009) and background selection (Stephan 2010) can all affect the detection of *F*_ST_ outliers. Furthermore, once candidate loci have been identified, there are few cases where their status has been confirmed in relation to phenotype or fitness impacts (Jensen *et al*. 2016). The exceptions are in cases where, like in our analyses, candidate loci were defined a priori (e.g. Colosimo *et al*. 2005; Hoekstra *et al*. 2006). We have sound biological reasons for looking at these candidates, we have followed them up in varied data sets and can confirm the outlier status of chemosensory genes as a category as well as some specific Or and Gr genes. It is now important to link these loci to behavioural differences between races, and to their assortative mating, and to examine the genomic context of these potential targets of selection.

R.K.B., C.M.S., J.F. and L.D. designed the study. J.F., L.D., D.H. and J.‐C.S. collected and reared aphids for DNA extraction. I.E., L.D., K.G. and R.T. generated the capture sequencing and GoldenGate SNP genotype data. I.E. and R.K.B. designed and performed the analyses. I.E. and R.K.B. wrote the article. All authors commented on draft versions of the manuscript. Authors declare no conflict of interests.

## Data accessibility

Capture sequencing reads are deposited in the EBI Sequence Read Archive (SRA) with project accession no. PRJEB6325. GoldenGate SNP genotypes are available in Table S4, table 1 (Supporting information), and the filtered set of capture sequencing SNPs used for PCAdapt analysis are available in Table S4, table 2 (Supporting information).

## Supporting information


**Appendix S1** Supplementary files 1a–1m.

**Table S1** 384 Target SNPs and flanking sequences for BeadXpress assay.

**Table S2** 179 SNPs retained from GoldenGate SNP genotyping assay, after processing and filtering results, with associated PCAdapt loadings, *P*‐values and *q*‐values.

**Table S3** (a) 7232 capture sequencing SNPs, with associated PCAdapt loadings, p‐values and q‐values. (b) Number of outlier and non‐outlier SNPs per gene (q<=0.05), and poisson probabilities of having this proportion of outliers in red: P < 0.05 Poisson probability of the observed or a greater number of SNP outliers given the number of SNPs in the gene and the overall proportion of outliers in grey shade: genes with 3 or more outlier SNPs in their sequence.

**Table S4** (a) GoldenGate SNP genotypes for 179 retained SNPs and 373 retained aphids. 0 = AA, 1 = AB, 2 = BB, 9 = missing data. (b) SNPs retained from capture sequencing dataset: input for PCAdapt analysis, 7232 SNPs in 116 aphids.

**Table S5** Outlier status for all 197 annotated chemosensory genes, in Smadja et al (2012), capture sequencing and GoldenGate SNP genotyping analyses, and comparison of these results.
