# Supplementary material for: Targeted re‐sequencing confirms the importance of chemosensory genes in aphid host race differentiation
Source: Mol Ecol. 2016 Sep 15;26(1):43–58. doi: 10.1111/mec.13818 (PMC6849616; doi:10.1111/mec.13818)

**Supplementary file 1a.** Aphid samples used in capture sequencing (from Duvaux *et al* (2015)).

| **Collection plant** | **Number of genotypes (after quality filtering)** |
| --- | --- |
| *Cytisus scoparius* | 17 |
| *Lotus corniculatus* | 12 |
| *Lotus pedunculatus* | 15 (13) |
| *Lathyrus pratensis* | 12 |
| *Medicago sativa* | 22 (21) |
| *Ononis spinosa* | 12 |
| *Pisum sativum* | 15 (14) |
| *Trifolium pratense* | 15 |

**Supplementary file 1b.** Scree plot from capture sequencing PCAdapt run (K=20 components) including *Lathyrus pratensis*-associated aphids.

**Supplementary file 1c.** plot of PC1 scores against PC2 scores from PCAdapt analysis of capture sequencing data, including *La. pratensis*-associated individuals. Points coloured according to species of collection plant.

**Supplementary file 1d.** histogram of PC1 squared loadings, from PCAdapt analysis of capture sequencing data, including *La. pratensis*-associated individuals.

**Supplementary file 1e.** Scree plot of capture sequencing analysis after removing *La.* *pratensis*-associated individuals.

**Supplementary file 1f.** aphid sampling details for GoldenGate SNP genotyping.

a) Sampling details

| **Population ID** | **Location** | **Code** | **Collection plant** | **# of aphids** |
| --- | --- | --- | --- | --- |
| CS.UK.1 | UK, Bristol | 5 | *Cytisus scoparius* | 20 |
| CS.UK.2 | UK, Sheffield | 43 | *Cytisus scoparius* | 56 |
| CS.EU.1 | France, Bugey | 46 | *Cytisus scoparius* | 7 |
| LAP.UK.1 | UK, Bristol | 1, 9, 16, 37, 41 | *Lathyrus pratensis* | 14 |
| LAP.UK.2 | UK, Peterborough | 19, 26 | *Lathyrus pratensis* | 8 |
| LAP.EU.1 | France, Bugey | 46 | *Lathyrus pratensis* | 14 |
| LOC.UK.1 | UK, Bristol | 3, 4, 5, 6, 7, 15, 16, 18 | *Lotus corniculatus* | 17 |
| LOC.UK.2 | UK, Peterborough | 18, 19, 22 | *Lotus corniculatus* | 7 |
| LOC.EU.1 | France, Bugey | 46 | *Lotus corniculatus* | 11 |
| LOP.UK.1 | UK, Bristol | 2, 5, 9, 15, 16, 41 | *Lotus pedunculatus* | 23 |
| LOP.UK.2 | UK, Peterborough | 19, 45 | *Lotus pedunculatus* | 22 |
| MS.UK.1 | UK, Blankney | 0 | *Medicago sativa* | 6 |
| MS.UK.2 | UK, Bristol | 8, 9 | *Medicago sativa* | 2 |
| MS.UK.3 | UK, Sheffield | 28 | *Medicago sativa* | 1 |
| MS.UK.4 | UK, Peterborough | 18 | *Medicago sativa* | 3 |
| MS.EU.1 | France, Mirecourt | 47 | *Medicago sativa* | 10 |
| MS.EU.2 | France, Volgelsheim | 47 | *Medicago sativa* | 12 |
| MS.EU.3 | Switzerland | 47 | *Medicago sativa* | 12 |
| OS.UK.1 | UK, Bristol | 7, 16, 41 | *Ononis spinosa* | 6 |
| OS.EU.1 | France, Bugey | 46 | *Ononis spinosa* | 28 |
| PS.UK.1 | UK, Bristol | 9, 10, 12, 13 | *Pisum sativum* | 13 |
| PS.UK.2 | UK, Sheffield | 28 | *Pisum sativum* | 12 |
| PS.UK.3 | UK, Peterborough | 18, 19, 25 | *Pisum sativum* | 5 |
| PS.EU.1 | France, Mirecourt | 47 | *Pisum sativum* | 10 |
| PS.EU.2 | France, Ranspach | 47 | *Pisum sativum* | 12 |
| TP.UK.1 | UK, Bristol | 2, 4, 5, 9, 12, 13, 17 | *Trifolium pratense* | 15 |
| TP.UK.2 | UK, Peterborough | 18, 26 | *Trifolium pratense* | 21 |
| TP.EU.1 | France, Mirecourt | 47 | *Trifolium pratense* | 12 |
| TP.EU.2 | Switzerland | 47 | *Trifolium pratense* | 12 |

b) Location codes

| **Code** | **Site** | **Latitude** | **Longitude** |
| --- | --- | --- | --- |
| 0 | British Chlorophyll, Blankney | 53.106553 | -0.463369 |
| 1 | Road verge at junction on B3130 to powder mill, Bristol | 51.37525 | -2.64747 |
| 2 | Gordano valley NNR – Moor lane, Bristol | 51.452055 | -2.817957 |
| 3 | Walton common (1), Bristol | 51.459114 | -2.827141 |
| 4 | Walton common (2), Bristol | 51.459542 | -2.824652 |
| 5 | Troopers Hill, Bristol | 51.455418 | -2.534561 |
| 6 | Whites Hill Allotments, Bristol | 51.45404 | -2.529228 |
| 7 | Plasters Green Meadow, Bristol | 51.346054 | -2.673247 |
| 8 | Winford Church, Bristol | 51.382308 | -2.657631 |
| 9 | Whaft lane (nr. Portishead), Bristol | 51.488478 | -2.742466 |
| 10 | Clevedon Allotment, Bristol | 51.442827 | -2.858367 |
| 12 | Chosen Hill farm, Bristol | 51.346145 | -2.593069 |
| 13 | Druid's farm (upper dre) – Pensford Lane, Bristol | 51.365384 | -2.569777 |
| 15 | path by disused mine at Pensford, Bristol | 51.361961 | -2.548596 |
| 16 | Chew lake (west), road side of B3114 at herons green, Bristol | 51.335183 | -2.639875 |
| 17 | Ashton court meadows, Bristol | 51.443763 | -2.658779 |
| 18 | Ring Haw, Peterborough | 52.574028 | -0.446282 |
| 19 | Wansford Pastures, Peterborough | 52.576485 | -0.412121 |
| 22 | Kingscliff meadow, Peterborough |  |  |
| 25 | Pea Growers Research Org, Peterborough | 52.592582 | -0.417138 |
| 26 | Road verge at Fortheringhay (towards Nassington), Peterborough | 52.531039 | -0.432346 |
| 28 | Rivlin valley allotments, Sheffield | 53.390273 | -1.521628 |
| 37 | Moreton lane - Chew valley lake (West coast) , Bristol | 51.322821 | -2.629684 |
| 41 | Gordano valley, Clapton Wick, Bristol | 51.45283 | -2.803962 |
| 43 | Ski village hill, Sheffield | 53.401589 | -1.480727 |
| 45 | Upwood, Peterborough | 52.441509 | -0.136496 |
| 46 | Details in Nouhaud *et al* (2014) |  |  |
| 47 | Details in Jaquiery *et al* (2012) |  |  |

**Supplementary file 1g.** Sampling locations for GoldenGate SNP genotyping dataset.

**Supplementary file 1h.** Scree plot from GoldenGate SNP genotyping PCAdapt run (K=20 components) including *Lathyrus pratensis*-associated aphids.

**Supplementary file 1i.** plot of PC1 scores against PC2 scores from PCAdapt analysis of GoldenGate SNP genotyping data, including *La. pratensis*-associated individuals. Points coloured according to species of collection plant.

**Supplementary file 1j.** Scree plot from PCAdapt analysis of GoldenGate SNP genotyping data after removing *La. pratensis*-associated individuals (K=20).

**Supplementary file 1k.** Re-classification and removal of incorrect genotypes prior to AMOVA analysis of GoldenGate SNP genotyping dataset.

| **Original name** | **Original race** | **Removed/reassigned** | **Reclassified race** |
| --- | --- | --- | --- |
| CS.UK.1.3 | *C. scoparius* | Removed |  |
| LAP.EU.1.14 | *L. pratensis* | PS.EU.3 | *P. sativum* |
| LAP.UK.1.14_REP6 | *L. pratensis* | Removed |  |
| LAP.UK.1.3 | *L. pratensis* | LOC.UK.1 | *L. corniculatus* |
| LOC.UK.1.16_REP | *L. corniculatus* | PS.UK.1 | *P. sativum* |
| LOP.UK.2.1_REP | *L. pedunculatus* | CS.UK.3 | *C. scoparius* |
| MS.EU.2.3 | *M. sativa* | Removed |  |
| MS.EU.3.1 | *M. sativa* | Removed |  |
| MS.UK.2.1 | *M. sativa* | LOC.UK.1 | *L. corniculatus* |
| MS.UK.3.1 | *M. sativa* | PS.UK.2 | *P. sativum* |
| OS.EU.1.26 | *O. spinosa* | LOC.EU.1 | *L. corniculatus* |
| OS.EU.1.30 | *O. spinosa* | Removed |  |
| OS.UK.1.5_REP6 | *O. spinosa* | Removed |  |
| PS.EU.1.6_REP | *P. sativum* | MS.EU.1 | *M. sativa* |
| TP.UK.2.15 | *T. pratense* | LAP.UK.2 | *L. pratensis* |
| TP.UK.2.20 | *T. pratense* | LAP.UK.2 | *L. pratensis* |

**Supplementary figure 1l.** Individuals from GoldenGate SNP genotyping analysis plotted according to score in principal components 1-6. To highlight the distinction between European and UK *Lo. corniculatus* individuals, European aphids are plotted with a cross. Aphids coloured by collection plant: black=*C. scoparius*, light blue=*La. pratensis*, green=*Lo. corniculatus*, light green=*Lo. pedunculatus*, red=*M. sativa*, orange=*O. spinosa*, blue=*P. sativum*, purple=*T. pratense.*

**Supplementary file 1m.** *F*_ST_ distributions of capture sequencing loci calculated according to groupings defined by the *L. pratensis* associated principal component, and by the other six principal components (PC1-6) (left to right, top to bottom).


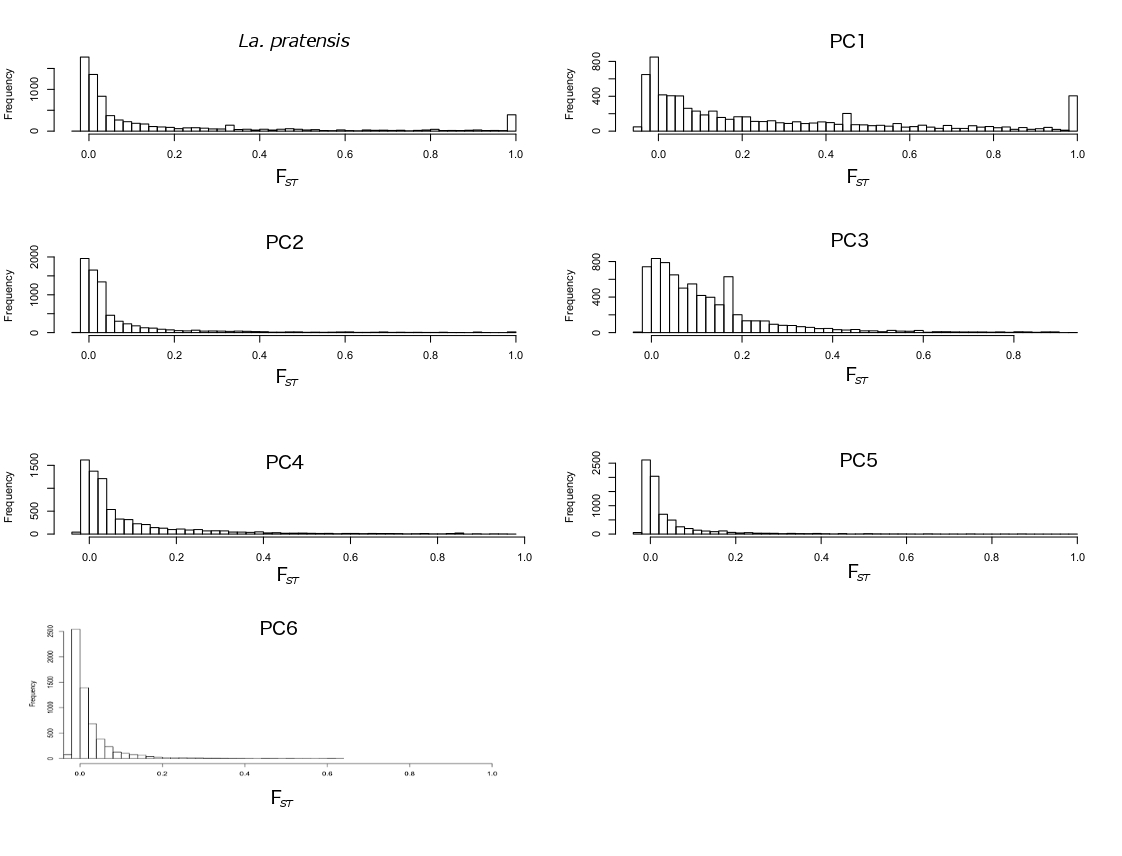

Supplement: Supplementary file 1 — Appendix S1 Supplementary files 1a–1m. [file MEC-26-43-s001.docx]
